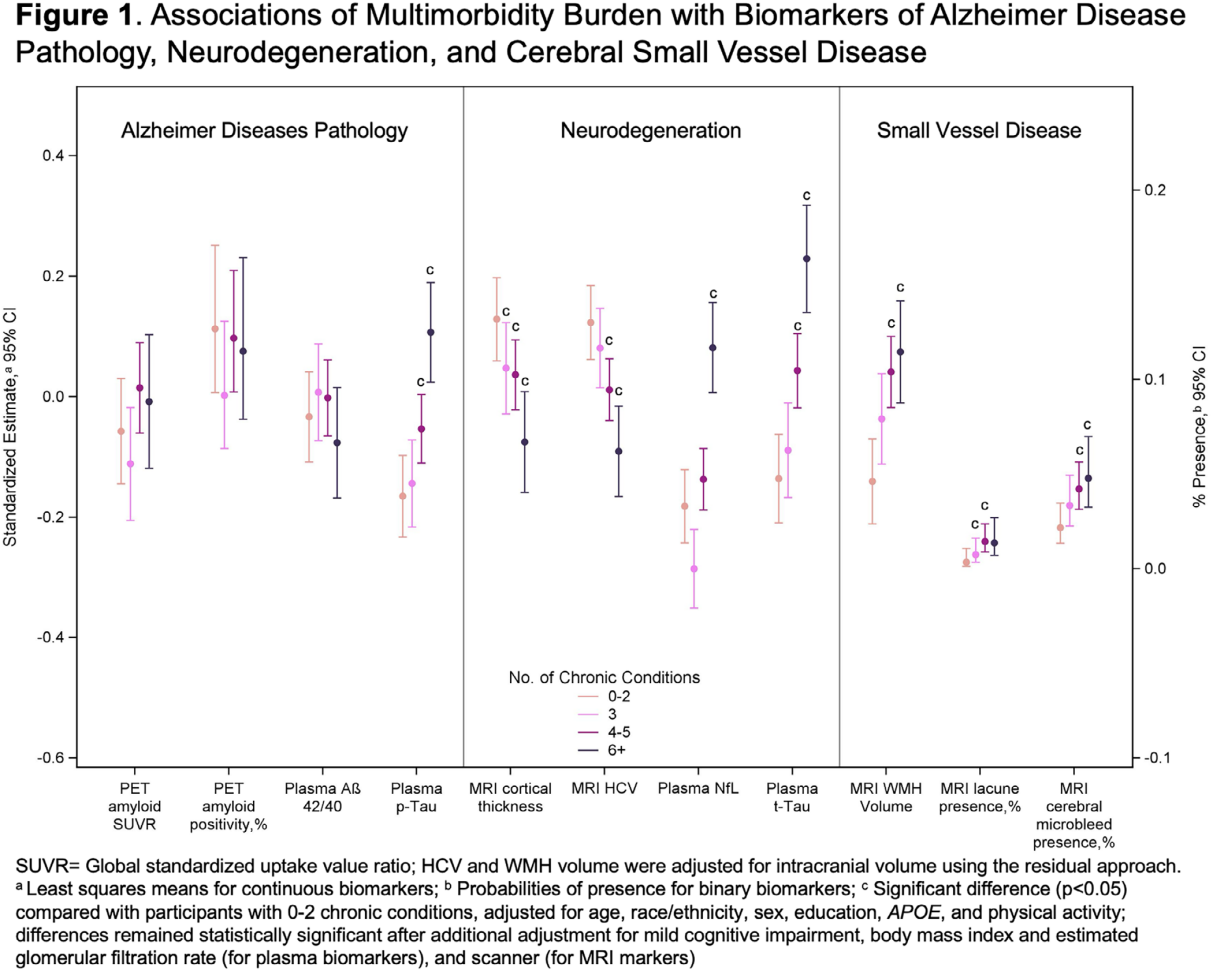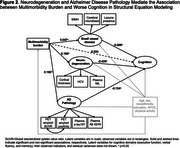# AT(N) Biomarkers Across Modalities in the Pathways Between Multimorbidity and Cognition

**DOI:** 10.1002/alz70861_108709

**Published:** 2025-12-23

**Authors:** Xiaqing Jiang, Sid E. O'Bryant, Robert A. Rissman, Leigh A. Johnson, Meredith N. Braskie, Kristine Yaffe

**Affiliations:** ^1^ University of California, San Francisco, San Francisco, CA USA; ^2^ University of North Texas Health Science Center, Fort Worth, TX USA; ^3^ Keck School of Medicine of the University of Southern California, Los Angeles, CA USA; ^4^ Mark and Mary Stevens Neuroimaging and Informatics Institute, Keck School of Medicine, University of Southern California, Los Angeles, CA USA

## Abstract

**Background:**

Multimorbidity, the coexistence of two or more chronic conditions, has been linked to cognitive aging and Alzheimer disease (AD) and AD‐related dementias, yet the mechanisms remain unclear. We investigated the associations of multimorbidity with cognition and mechanisms through AD pathology, neurodegeneration and cerebral small vessel disease (SVD).

**Method:**

We cross‐sectionally studied a diverse cohort of 3,176 dementia‐free participants (64% women, 37% Hispanic, 37% non‐Hispanic White, 26% Black) aged ≥50 years (mean age 64.6 ± 8.5) in the Health and Aging Brain Study: Health Disparities. Multimorbidity burden was assessed by counting the total number of chronic conditions out of 22 conditions identified through objective measures, laboratory values, medical history, and self‐reports. A latent factor score for overall cognition was estimated using confirmatory factor analysis and neuropsychological tests. Biomarkers assessed included AD pathology (PET amyloid, plasma β‐amyloid [Aβ] 42/40 and phosphorylated tau‐181 [p‐tau]), neurodegeneration (cortical thickness, hippocampal volume [HCV], and plasma neurofilament light [NfL] and total tau), and cerebral SVD (MRI white matter hyperintensities [WMH], cerebral microbleeds, and lacunes). We used linear and logistic regression to assess the associations of multimorbidity burden with continuous and dichotomous biomarkers, respectively. Structural equation modeling (SEM) was used to assess the association of multimorbidity with cognition and mediating pathways.

**Result:**

After adjusting for age, sex, race/ethnicity, education, *APOE* ε4, and physical activity, a greater multimorbidity burden (per 3 additional conditions) was associated with worse overall cognition (standardized β =‐0.074, 95%CI ‐0.104 to ‐0.043) and biomarkers indicative of greater AD pathology, neurodegeneration, and SVD (Figure 1). The direct association between multimorbidity and cognition was attenuated (standardized‐β=0.024, 95%CI ‐0.023 to 0.071) after accounting for the mediating pathways (Figure 2). SEM showed that the association between multimorbidity and worse cognition was primarily mediated by neurodegeneration (74%, standardized‐β =‐0.072, 95%CI ‐0.109 to ‐0.035) and partially by AD pathology (22%, standardized‐β =‐0.022, 95%CI ‐0.038 to ‐0.005), after multivariable adjustment.

**Conclusion:**

Among dementia‐free individuals, greater multimorbidity burden was associated with biomarkers indicative of greater AD pathology, neurodegeneration, and cerebral SVD. Multimorbidity may contribute to cognition via both neurodegeneration and AD pathology pathways.